# Vector-Borne Agents in Species of Silky Anteater (*Cyclopes* Gray, 1821) from South America

**DOI:** 10.3390/pathogens14070718

**Published:** 2025-07-19

**Authors:** Pedro Henrique Cotrin Rodrigues, João Paulo Soares Alves, Flávia Regina Miranda, Cesar Rojano, Júlia Angélica Gonçalves Silveira

**Affiliations:** 1 Laboratório de Protozoologia Veterinária, Departamento de Medicina Veterinária Preventiva, Escola de Veterinária, Universidade Federal de Minas Gerais, Belo Horizonte 31270-901, Minas Gerais, Brazil; pedro.cotrinr@gmail.com (P.H.C.R.); joaopsavet@gmail.com (J.P.S.A.); 2 Departamento de Ciência Animal, Universidade Estadual de Santa Cruz, Ilhéus 45662-900, Bahia, Brazil; frmiranda@uesc.br; 3 Fundacion Cunaguaro, Yopal 850001, Casanare, Colombia; c.rojanob@gmail.com

**Keywords:** hemopathogens, xenarthra, Brazil, Peru and Colombia, free-living wildlife

## Abstract

*Cyclopes*, the smallest of all known anteaters, has an insectivorous diet and is arboreal, rarely descending to the ground. There are scarce reports on diseases and pathogenic agents affecting this taxon. Hemopathogens are pathogenic agents that inhabit the blood of various vertebrate species. Protozoa such as *Trypanosoma* spp., *Leishmania* spp., *Hepatozoon* spp., and members of the order Piroplasmida, as well as hemoplasmas and Rickettsial bacteria of the genera *Anaplasma* and *Ehrlichia*, are among the most important in this group. The transmission of these pathogens generally occurs through arthropod vectors, which act as intermediate hosts. In addition, infections caused by hemopathogens can have adverse effects on host health, contributing to population declines in susceptible species. This study investigated infection by protozoa and hemotropic bacteria in blood samples from free-ranging silky anteaters from Brazil, Peru, and Colombia using molecular detection methods. Sixteen samples were obtained during expeditions conducted in these countries. DNA was extracted from blood samples, and PCR assays were performed to detect parasites from the order Piroplasmida, *Hepatozoon* spp., trypanosomatid agents including *Leishmania* spp., *Trypanosoma evansi*, *T. cruzi*, and *T. vivax*, as well as hemotropic bacteria of the genera *Ehrlichia*, *Anaplasma*, and *Mycoplasma* sp. Nucleotide sequencing was performed on positive samples. Of the total samples analyzed, 62.5% (10/16) tested positive for hemotropic *Mycoplasma*, 50% (8/16) for *T. evansi*, and 6.2% (1/16) for *T. cruzi*. There is a significant gap in knowledge regarding the diversity of hemopathogens affecting the genus *Cyclopes*, and future studies are needed to understand how these infections may impact the health of individuals.

## 1. Introduction

Silky anteaters are mammals belonging to the order Pilosa, family Cyclopedidae. *Cyclopes didactylus* Linnaeus, 1758, was historically considered the only species within the family Cyclopedidae. However, a taxonomic revision of the genus *Cyclopes*, using an integrative approach with morphological, morphometric, biogeographic, and molecular data, redefined its taxonomy. The authors demonstrated that four previously described species are considered valid: *C. didactylus* Linnaeus, 1758, *C. ida* Thomas, 1900, *C. catellus* Thomas, 1928, and *C. dorsalis* Gray, 1865. Additionally, three new species were described: *C. rufus* Miranda, 2017, *C. thomasi* Miranda, 2017, and *C. xinguensis* Miranda, 2017 [1].

*Cyclopes* spp. inhabit tropical forests at elevations above 1500 m across the American continent [2,3], with a distribution ranging from southern Mexico to South America [4,5]. This genus represents the smallest of all extant anteaters, with adults measuring, on average, between 15 and 35 cm in length [6,7] and rarely exceeding 400 g in body weight [5,8]. These species exhibit solitary, predominantly arboreal, and strictly insectivorous behavior, rarely descending to the ground [9]. Primarily nocturnal, they rest in the tree canopy during the day, typically not using the same tree for more than two consecutive days [6]. These ecological and behavioral characteristics contribute to this being the least studied species within the superorder Xenarthra [10].

The main threats identified for this taxon include agricultural expansion, deforestation, habitat fragmentation, and illegal capture and trade [11]. According to the IUCN Red List of Threatened Species, there is an urgent need for further research on the taxonomy, genetics, and basic ecology of this species to support its conservation [11]. Additionally, there are very few reports regarding diseases and pathogens affecting the genus *Cyclopes* [12].

Hemopathogens are pathogenic agents that inhabit the blood of various vertebrate species. Protozoa such as *Trypanosoma* spp. Gruby, 1843, *Leishmania* spp. Ross, 1903, *Hepatozoon* spp. Miller, 1908, and members of the order Piroplasmida Wenyon, 1926, as well as hemoplasmas Nowak, 1929, and Rickettsial bacteria of the genera *Anaplasma* Theiler, 1910, and *Ehrlichia* Moshkovski, 1945, are among the most significant in this group [13,14]. Transmission of these pathogens generally occurs through arthropod vectors that act as intermediate hosts, including triatomines, ticks, mosquitoes, and sandflies [15,16,17]. Infection by hemopathogens can negatively affect host health, potentially contributing to population declines in vulnerable species [18,19].

There is a significant knowledge gap regarding the diversity of hemopathogens affecting the genus *Cyclopes*, which hinders the assessment of their ecological and health impacts. Understanding the occurrence and distribution of these pathogens in understudied species is essential for the implementation of effective conservation and management strategies. In this context, the aim of this study was to investigate the presence of protozoan and hemotropic bacterial infections in blood samples from free-ranging *Cyclopes* spp. from Brazil, Peru, and Colombia using molecular detection methods.

## 2. Materials and Methods

### 2.1. Sampling

Capture and sampling permits were granted by the Chico Mendes Institute for Biodiversity Conservation—ICMBio (SisBio permit numbers: 125811, 125813, and 133813).

Sampling was conducted between 2007 and 2016 during field expeditions in Brazil, Peru, and Colombia. Using active search methods, individuals were captured in their natural habitat during daylight hours and individually placed in cotton bags for administration of the anesthetic protocol. An intramuscular anesthesia protocol was applied, consisting of ketamine hydrochloride (8.0 mg/kg; Ketalar^®^, 50 mg/mL; Pfizer Laboratorie, São Paulo, Brazil) combined with midazolam (0.5 mg/kg; Dormonid^®^, 5 mg/5 mL; Roche Laboratorie, São Paulo, Brazil). The geographic location of each capture was recorded.

Immediately after anesthesia, approximately 2 mL of blood was collected via puncture of the cephalic, caudal, or femoral vein using 0.7 × 30 mm needles and a 5 mL syringe. The blood was stored in 3 mL EDTA tubes (BD Vacutainer^®^, São Paulo, Brazil) and maintained by refrigerating in a styrofoam container with ice throughout the field procedures, up to the time of laboratory analysis. Additionally, a thorough inspection of the animal’s body surface was performed, limited to macroscopic visual examination, in order to detect the presence of ectoparasites. During anesthesia, vital parameters were monitored, including heart rate and rhythm, respiratory rate, rectal temperature, and oxygen saturation (measured via pulse oximetry). In addition, complementary procedures were performed during immobilization, such as microchip implantation, tattooing, weighing, morphometric measurements, sex identification, age estimation (determined based on body mass, density of hair, and size), and biological sample collection. Individuals were kept under monitoring until full recovery from anesthesia (average recovery time of 40 min). Once fully recovered, the animals were released at the exact site of capture.

**Figure 1:**
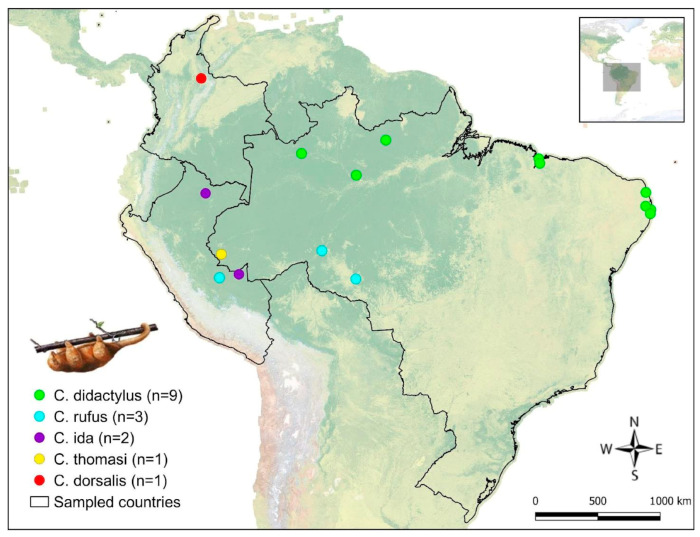
Map showing a partial representation of South America indicating the locations where animals were sampled. Circles represent the different sampled species according to their capture sites. Black lines represent the borders of the sampled countries (Brazil, Peru, and Colombia). Illustration of *C. didactylus* by Eisenberg and Redford [8]. The map was generated using QGIS (v. 3.32.0, 2023) with WGS-84 as the geographic reference system [20].

A total of 16 samples were collected. The identification of the samples and the geographical distribution of *Cyclopes* species included in this study are summarized in Table 1 and illustrated in Figure 1. Species identification was based on morphological and molecular methods, as described in a previous study [1].

### 2.2. Molecular Analysis

DNA was extracted from blood samples using the Wizard^®^ Genomic DNA Purification Kit (Promega, Madison, WI, USA), following the manufacturer’s instructions. To verify the extraction quality, the integrity of the obtained DNA, and the presence of potential PCR inhibitors, the extracted samples were tested for the presence of the gene encoding glyceraldehyde-3-phosphate dehydrogenase (*gapdh*), a housekeeping gene in mammals [21].

Nested PCR and conventional PCR assays were performed to detect the presence of pathogens belonging to the order Piroplasmida, *Hepatozoon* spp., *Ehrlichia* spp., *Anaplasma* spp., hemotropic *Mycoplasma*, and trypanosomatids, including *Leishmania* spp., *Trypanosoma evansi* Steel, 1885, *T. cruzi* Chagas, 1909, and *T. vivax* Ziemann, 1905.

For all PCR assays, the reaction mixture for the first round contained 7.5 µL of GoTaq^®^ Green Master Mix (Promega, Madison, WI, USA), 0.6 µL of a mixed primer solution (10 mM), and 5.4 µL of nuclease-free water. A volume of 1.5 µL of total DNA was added to the reaction mixture to achieve a final volume of 15 µL. For nested PCR assays, 1.5 µL of the amplified product from the first reaction was used as the DNA template for the second reaction. The sets of primers used for pathogen detection were applied with a touchdown PCR protocol, programmed as previously described. All primers, nucleotide sequences, target genes, and expected amplicon sizes used in this study are detailed in Table 2. In all reactions, ultrapure sterile water (Life Technologies^®^, Carlsbad, CA, USA) was used instead of template DNA as a negative control.

As positive controls, DNA extracted from different sources was used. For *Ehrlichia* spp., DNA was extracted from the whole blood of domestic dogs (*Canis familiaris,* Linnaeus, 1758) experimentally infected with *Ehrlichia canis*, Donatien and Lestoquard, 1935 (Jaboticabal strain) [40]. DNA from *Anaplasma marginale* Theiler, 1910 (UFMG1 strain), was obtained from the whole blood of a calf experimentally infected [41]. For the nested PCR targeting Piroplasmida/*Hepatozoon* spp., DNA was extracted from a calf experimentally infected with *Babesia bovis* Babes, 1888 (BbovMG strain), and *B. bigemina*, Smith and Kilborne, 1893 (BbigMG strain) [42]. For *Anaplasma phagocytophilum* Foggie, 1949, and other granulocytic agents from the family Anaplasmataceae, DNA from IDE8 tick cell cultures infected with *A. phagocytophilum* (isolated from a German dog) was kindly provided by Dr. Erich Zweygarth (Institut für Vergleichende Tropenmedizin und Parasitologie, Ludwig Maximilians Universität München). For *Leishmania* spp. testing, DNA from the reference strain *L. infantum* Nicolle, 1908 (MCAN/BR/2002/BH400), provided by the World Health Organization (WHO) and maintained in the cryobank of the Leishmania Biology Laboratory, was kindly provided by Prof. Maria Norma Melo (ICB/UFMG, Brazil). For *Trypanosoma evansi*, *T. vivax*, and hemotropic *Mycoplasma*, whole blood samples from naturally infected South American coatis (*Nasua nasua* Linnaeus, 1766), cattle (*Bos taurus* Linnaeus, 1758), and domestic cats (*Felis catus* Linnaeus, 1758), respectively, were used [43,44,45]. For *T. cruzi*, DNA was extracted from experimentally infected mice, kindly provided by the *T. cruzi* and Chagas Disease Biology Laboratory—ICB/UFMG.

All reactions were performed using the same thermal cycler—Applied Biosystems MiniAmp Thermal Cycler (Thermo Fisher Scientific, Waltham, MA, USA). PCR products were separated by electrophoresis on 1% agarose gels (40 min, 100 V), stained with GelRed™ (Biotium, Hayward, CA, USA), and visualized under ultraviolet light.

### 2.3. Genetic Sequencing

For sequencing, the PCR-positive products were purified using the QIAquick PCR Purification Kit (Qiagen Biotecnologia Brasil, São Paulo, Brazil) according to the manufacturer’s instructions. The purified amplicons were sequenced using the Sanger method [46] on an ABI3130 Genetic Analyzer (Applied Biosystems, Life Technologies, Carlsbad, CA, USA) with the BigDye^®^ Direct Cycle Sequencing Kit v3.1 (Applied Biosystems) and the POP-7™ polymer as the separation matrix, employing the same primers used in the PCR reactions.

The forward and reverse sequences obtained were aligned, edited, and analyzed using MEGA (Molecular Evolutionary Genetics Analysis) version 11. The identity of each sequence was confirmed through alignment by homology with sequences available in GenBank using the BLASTn software [47].

### 2.4. Phylogenetic Analysis

Phylogenetic trees were constructed using MEGA software, version 11. Multiple sequence alignments were performed with ClustalW, integrated into the platform, using sequences previously submitted to GenBank. The most appropriate evolutionary models were selected based on the lowest scores of the Bayesian Information Criterion (BIC) and the corrected Akaike Information Criterion (AICc). Representative sequences were included as an outgroup in the analysis. The phylogenetic tree was generated using the maximum likelihood (ML) method, based on the obtained alignments [48,49,50]. The robustness of the tree topology was tested through 1000 bootstrap replicates [51].

## 3. Results

**Figure 2:**
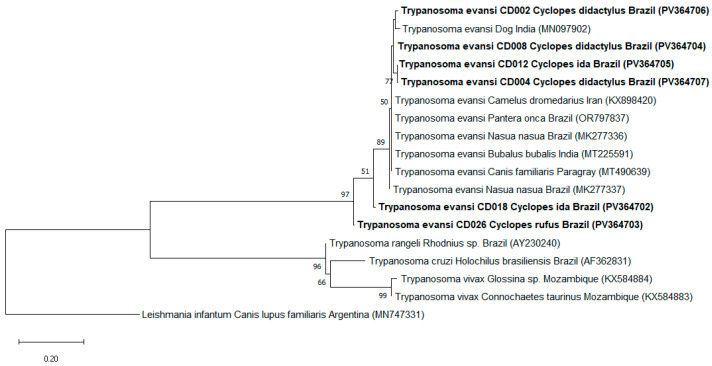
Phylogenetic tree based on a 219 bp alignment of the *ITS-1* gene of *Trypanosoma evansi*, including 18 nucleotide sequences, constructed using the maximum likelihood (ML) method and the Jukes–Cantor (JC) evolutionary model. Numbers at the tree branches indicate bootstrap values based on 1000 replicates; values below 40 are not shown. The scale bar represents evolutionary distance. Sequences detected in the present study are highlighted in bold, with their GenBank accession numbers shown in parentheses. *Leishmania infantum* was used as the outgroup.

All 16 DNA samples analyzed tested positive for the mammalian endogenous gene (*gapdh*). Subsequent molecular tests detected the presence of *T. evansi* (*ITS-1* gene) in 50% (8/16) of the analyzed samples, including six samples from Brazil (four *C. didactylus*, one *C. thomasi*, and one *C. rufus*) and two from Peru (two *C. ida*). All eight samples were also positive for the amplification of the 18S rRNA gene of Kinetoplastida. Positive samples were subjected to genetic sequencing; however, only six *ITS* gene products yielded readable sequences (GenBank accession numbers: PV364702 to PV364707). BLASTn analyses showed percent identity ranging from 92% to 98.6% with *T. evansi* sequences from different hosts, including *N. nasua* (MK277343; MK277341) from Brazil, camel (*Camelus* spp. Linnaeus, 1758) (MH595480) from Iraq, and water buffalo (*Bubalus bubalis* Linnaeus, 1758) (MT225591) from India. Moreover, the maximum likelihood (ML) analysis based on the *ITS-1* gene (219 bp), using the Jukes–Cantor (JC) model, clustered the *T. evansi* sequences detected in this study with sequences of the species sampled from *C. familiaris*, *Camelus dromedarius*, *Panthera onca* Linnaeus, 1758, *N. nasua*, and *B. bubalis* collected in India, Iran, Brazil, and Paraguay (Figure 2).

**Figure 3:**
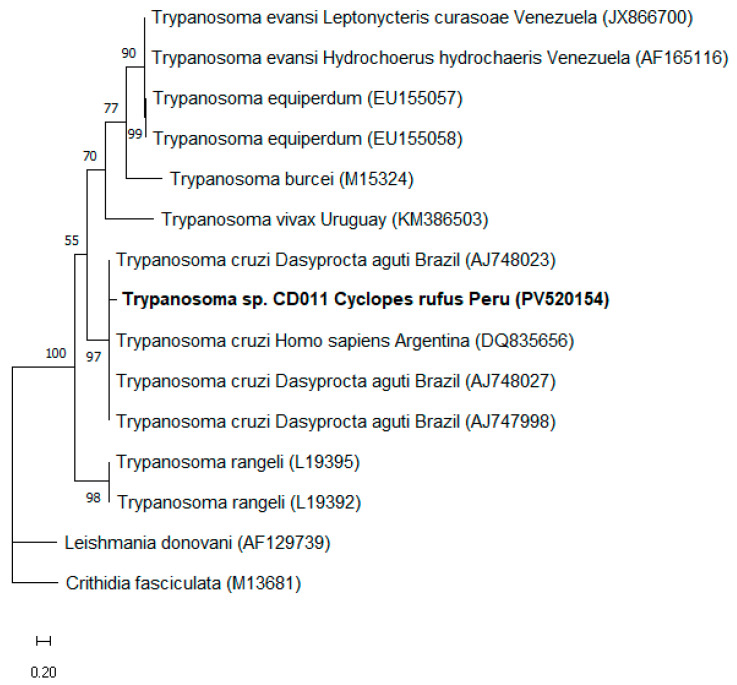
Phylogenetic tree based on a 300 bp alignment of the *kDNA* gene of *Trypanosoma* sp., including 16 nucleotide sequences, constructed using the maximum likelihood (ML) method and the Tamura 3-parameter (T92) evolutionary model. Numbers at the tree branches indicate bootstrap values based on 1000 replicates. The scale bar represents evolutionary distance. The sequence detected in the present study is highlighted in bold, with its GenBank accession number shown in parentheses. *Leishmania donovani* and *Crithidia fasciculata* were used as outgroups.

Additionally, 6.2% (1/16) of the individuals analyzed, represented by one specimen of *C. rufus* from Peru, tested positive for *T. cruzi/T. rangeli* (*kDNA* gene). Genetic sequencing of the amplified fragment (accession PV520154) revealed 87.6% identity with *T. cruzi* sequences previously isolated from agouti *(Dasyprocta aguti* Linnaeus, 1766) (AJ748023, AJ748027, AJ747998) and *Homo sapiens* Linnaeus, 1758 (DQ835656), from Brazil and Argentina, respectively. The sequencing reaction was performed in triplicate using both forward and reverse primers, and the resulting electropherograms were used to generate a consensus sequence, which consistently yielded similar identity values across all replicates. Pairwise alignment with the reference sequence AJ748023 showed 41 nucleotide differences over 329 base pairs (query cover 96%), supporting the calculated identity percentage. This same sample also tested positive for the 18S rRNA and *kDNA* genes of Kinetoplastida; however, the sequences obtained were not considered of sufficient quality for conclusive analyses. Phylogenetic inference performed using ML, based on kDNA gene sequences (307 bp) and the Tamura three-parameter (T92) substitution model, consistently clustered the *Trypanosoma* sp. sequence detected in this study within the same clade as *T. cruzi* sequences obtained from different hosts in Brazil and Argentina (Figure 3). Although the sequence clustered robustly with *T. cruzi* (bootstrap = 97%), it was conservatively labeled as *Trypanosoma* sp. in the phylogenetic tree due to the moderate identity value and relatively short fragment length. This clustering showed high statistical support, indicating a robust evolutionary relationship between the sequence obtained and previously described *T. cruzi* strains.

**Figure 4:**
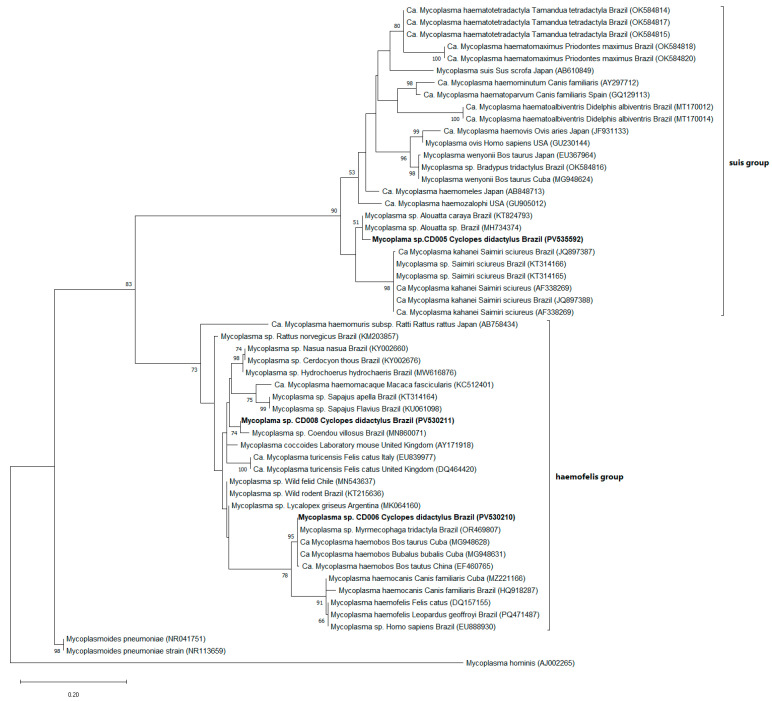
Phylogenetic tree based on a 584 bp alignment of the 16S rRNA gene of *Mycoplasma* spp., including 56 nucleotide sequences, constructed using the maximum likelihood (ML) method and the Tamura 3-parameter model with gamma distribution (T93+G). Numbers at the tree branches indicate bootstrap values based on 1000 replicates; values below 50 are not shown. The scale bar represents evolutionary distance. The sequences detected in the present study are highlighted in bold, with their GenBank accession numbers shown in parentheses. *Mycoplasma hominis* was used as the outgroup.

The screening PCR based on the 16S rRNA gene (600 and 193 bp) of hemoplasmas revealed a positivity rate of 62.5% (10/16), including seven individuals from Brazil (five *C. didactylus*, one *C. rufus*, and one *C. thomasi*) and three from Peru (one *C. rufus* and two *C. ida*). Additionally, 5 out of the 10 samples positive for the 16S rRNA gene were also positive for the 23S rRNA gene (800 bp) (4 *C. didactylus* from Brazil and 1 *C. ida* from Peru). PCR products with strong band intensity were selected for genetic sequencing; however, satisfactory results were obtained for only three 16S rRNA gene sequences (accessions PV530210, PV530211, PV535592). BLASTn analyses revealed high identity between one of the obtained sequences (CD005) and hemotropic *Mycoplasma* sp. previously detected in arboreal Neotropical primates of the genus *Alouatta* spp. Lacépède, 1799, in Brazil (KT824793, JQ897386, MH734376, MH734374), with percentage identity ranging from 97.7% to 98.5%. Furthermore, this sequence showed 95.4% to 96.1% identity with ‘*Candidatus* Mycoplasma kahanei’, previously described in *Saimiri sciureus* Linnaeus, 1758, another arboreal primate from Brazil (JQ897388) and the United States (AF338269). Another sequence (CD006) showed 99.4% to 99.8% identity with *Mycoplasma* sp. and ‘*Ca*. Mycoplasma haemobos’, previously detected in giant anteater *(Myrmecophaga tridactyla* Linnaeus, 1758) (OR469807) from Brazil and bovids (MG948628, MG948630, MG948631) sampled in Central America. The third and final readable sequence analyzed (CD008) showed 97.9% to 98.4% identity with *Mycoplasma* sp. isolated from *Coendou villosus* Coendou Lacépède, 1799, in Brazil (MN860071) and *Mycoplasma* lineages detected in *Lycalopex griseus* Gray, 1837 (MK064160), wild felids (MN543637), and wild rodents (KT215636). Phylogenetic inference performed using ML, based on the 16S rRNA gene and the Tamura three-parameter model with gamma distribution (T92+G), grouped the *Mycoplasma* spp. sequences detected in this study into two main clades, which, according to the authors of [52], can be divided into the *haemofelis* group (*n* = 2) and the *suis* group (*n* = 1), both supported by high bootstrap values. One sample from the *suis* group (CD005) clustered within the same clade as hemoplasmas previously detected in arboreal primates, with phylogenetic proximity to the ‘*Ca*. Mycoplasma kahanei’ clade. Of the two samples grouped in the *haemofelis* group, one (CD006) showed high similarity with *Mycoplasma* sequences isolated from *M. tridactyla*, both positioned within the same clade as ‘*Ca*. Mycoplasma haemobos’. Meanwhile, sample CD008 showed a closer phylogenetic relationship to *Mycoplasma* spp. detected in *C. villosus*, a Brazilian mammal that also has arboreal habits (Figure 4).

Among the 14 individuals positive for at least one of the pathogens investigated, 35.7% (5/14) presented co-infections involving two agents. Of these, 80% (4/5) corresponded to co-infection with hemotropic *Mycoplasma* and *T. evansi*, detected in two *C. ida* individuals from Peru, one *C. thomasi*, and one *C. didactylus* from Brazil. The remaining co-infection, representing 20% (1/5) of the cases, involved *T. cruzi* and hemotropic *Mycoplasma* in a *C. rufus* individual sampled in Peru. No co-infections involving more than two pathogens were identified. In the present study, no samples tested positive for pathogens of the order Piroplasmida, *Hepatozoon* sp., agents of the family Anaplasmataceae, or other trypanosomatids such as *Leishmania* spp. and *T. vivax* (Table 3). Additionally, no ectoparasites were observed on any of the body regions examined, based on macroscopic visual inspection.

## 4. Discussion

To the best of our knowledge, this is the first molecular detection of *Trypanosoma evansi* and hemotropic *Mycoplasma* in newly described species of the genus *Cyclopes*. These species are strictly arboreal and rarely descend to the ground, a behavior that can hinder their observation, capture, and the collection of blood samples. This fact makes the present study particularly relevant for advancing knowledge about the health status of this genus. Additionally, the taxonomic description of different *Cyclopes* species is relatively recent, and studies focused on these newly described species are still scarce.

There are very few studies on hemopathogens in silky anteaters. Between 1936 and 1938, Deane [53] examined 12 specimens of *C. didactylus* from the state of Pará, Brazil, using blood smears, tissue imprints, in vitro culture, and inoculation into other animals, but found no evidence of hemoflagellate infection. Later, Lainson et al. [54], using similar methods combined with xenodiagnosis, also failed to detect Trypanosomatidae in *C. didactylus* from the same region. However, when parasitemia is low, false-negative results may occur. The first isolation of a trypanosomatid identified as *T. cruzi* in *C. didactylus* was reported by Miles et al. [55] during a survey of wild mammals in the state of Pará. Since then, there have been no further records of studies aimed at detecting this or other pathogens in this genus.

Infections caused by Trypanosomatidae in xenarthrans have been reported in the state of Pará, Brazil, showing mixed infections with *T. cruzi*, *T. rangeli* Tejera, 1920, and *L. infantum* in the lesser anteater (*Tamandua tetradactyla* Linnaeus, 1758) [56]. Another study highlighted this species as an important reservoir for *T. rangeli* and an efficient vector of the parasite in the Brazilian Amazon region [57]. Additionally, *T. cruzi* was detected in two six-banded armadillos (*Euphractus sexcinctus* Linnaeus, 1758), one *T. tetradactyla*, and one nine-banded armadillo (*Dasypus novemcinctus* Linnaeus, 1758) in the Brazilian Pantanal. This was the first report of *T. tetradactyla* infected with *T. cruzi* in the Pantanal region, with positivity confirmed through hemoculture, showing high levels of parasitemia during the sampling period. The authors concluded that *T. tetradactyla* are important hosts for *T. cruzi* in the studied area [58]. However, the epidemiological relevance of *Cyclopes* spp. as hosts for this pathogen remains unclear.

*Trypanosoma cruzi* is a hemoflagellate responsible for Chagas disease, transmitted by triatomine bugs. Some authors have reported that the most favorable climatic conditions for the presence of triatomines include humid mesothermal climates, as higher temperatures promote a greater geographical dispersion of wild vectors [59,60]. These climatic conditions are present in the sampling areas, which may explain the detection of the pathogen in a blood sample from *C. rufus* collected in Peru. In that country, Chagas disease surveillance has been mandatory nationwide since 1999. Despite this, the distribution of cases remains irregular. Among the regions reporting Chagas disease in Peru, Ucayali—the origin of the positive silky anteater—accounts for 3.2% of the reported cases, reinforcing the circulation of the parasite in the region and, consequently, the possibility of infection in wild animals [61].

*Trypanosoma evansi* is a member of the Trypanosomatidae family that can parasitize a wide range of domestic and wild mammalian hosts and causes a trypanosomiasis known as ‘surra.’ In the Americas, the transmission of *T. evansi* occurs predominantly through mechanical means, via the blood of infected animals transmitted by hematophagous dipterans such as members of the Tabanidae family. Additionally, it can be transmitted vertically, iatrogenically, through the ingestion of infected prey, or via the action of hematophagous bats (*Desmodus rotundus* Geoffroy, 1810) [62,63,64]. It is a protozoan parasite found in both intravascular and extravascular fluids [65] and, in susceptible animals, can cause clinical manifestations. The infiltration and dissemination of *T. evansi* into the central nervous system have been reported, and neurological signs are common in horses [64,66]. Furthermore, animals subjected to stress, malnutrition, or pregnancy are more susceptible to the disease [66].

This study reports the presence of *T. evansi* parasitizing silky anteaters in tropical areas of Brazil and Peru, where this parasite was likely introduced during the 16th century with horses or mules brought by Spanish conquistadors [67,68]. Brazilian wild animals, such as deer, capybaras, and coatis, can become infected and may develop disease, including death, but may also act as reservoirs. However, the different habitats and behavioral patterns of wild and domestic hosts suggest that there are still unknown factors underlying the transmission cycles of *T. evansi* [36,63,69].

Little is known about the role of Xenarthra in the epidemiology of *T. evansi* infection. In a study conducted by Herrera et al. [63], which evaluated the infection by this agent in domestic and wild species in the Pantanal region of Brazil, PCR detection was reported in one (one out of eight) armadillo (*Euphractus* sp.). Santos et al. [58] investigated the role of different host species in the transmission cycles of *Trypanosoma* spp. in a central area of the Brazilian Pantanal. Among Xenarthra, they found PCR-positive results for *T. evansi* in two (2/29) six-banded armadillos *(Euphractus sexcinctus)*.

Bacteria of the genus *Mycoplasma*, also known as hemoplasmas, can have significant implications for animal and human health [70,71,72]. These epierythrocytic microorganisms adhere to the surface of erythrocytes, and although generally considered to have low pathogenicity, they can cause clinical disease under conditions of immunosuppression or co-infection [18,73]. While vector-borne transmission by hematophagous arthropods is widely recognized, there is evidence supporting alternative transmission routes, such as transplacental transmission and exposure to the blood of infected animals during aggressive interactions [74,75,76,77]. Among Xenarthra, specific behaviors such as fights between anteaters (*M. tridactyla*) [78] and the shared use of burrows by armadillos [79] may facilitate both direct and vector-borne transmission of these hemopathogens, representing important ecological aspects to be considered in surveillance and conservation studies of these taxa. However, in the genus *Cyclopes*, given its strictly solitary nature, other transmission routes should be prioritized when evaluating health risks.

Recent studies have reported the occurrence of hemoplasmas in members of the order Xenarthra. De Oliveira et al. [80] reported the detection of these microorganisms in anteaters (*M. tridactyla* and *T. tetradactyla*), sloths (genera *Bradypus* Linnaeus, 1758, and Choloepus Illiger, 1811), and armadillos (*Priodontes maximus* Kerr, 1792, *E. sexcinctus*, and *D. novemcinctus*) sampled from different regions of Brazil. Notably, the study also described, for the first time, the presence of a 16S rRNA sequence with high identity (99.7%) to *Mycoplasma wenyonii* in pale-throated sloth *(Bradypus tridactylus* Linnaeus, 1758), in addition to the molecular characterization of two novel taxa, designated as ‘*Ca*. Mycoplasma haematotetradactyla’ and ‘*Ca*. Mycoplasma haematomaximus’.

Another study conducted by Sada et al. [19] also reported a considerably high positivity rate (52%) in armadillos (*E. sexcinctus*, *P. maximus*, and *D. novemcinctus*) and anteaters (*M. tridactyla* and *T. tetradactyla*) from the Southeast and Midwest regions of Brazil. The authors detected the presence of a hemoplasma phylogenetically related to ‘*Ca*. Mycoplasma haematomaximus’, previously described in *P. maximus* in the state of Mato Grosso do Sul. It is noteworthy that most of the animals sampled by Sada et al. [19] originated from Mato Grosso do Sul, a region characterized by high biodiversity and vector density.

These findings, together with the study by De Oliveira et al. [80], contribute significantly to advancing knowledge about the genetic diversity and distribution of hemoplasmas in free-ranging Xenarthra, highlighting the importance of these hosts in the ecology of these agents. However, the genus *Cyclopes* remains the least studied among Xenarthra, lacking investigations that assess the occurrence and diversity of hemoplasmas in these species and their implications for the health of this taxon.

The present study revealed a high positivity rate for hemoplasmas (62.5%) in individuals of the genus *Cyclopes* sampled in Brazil and Peru, representing the first report of the detection of this agent in this group. Phylogenetic analysis revealed a close relationship between two sequences obtained in this study (CD005 and CD008) and *Mycoplasma* spp. previously detected in *C. villosus* and neotropical arboreal primates of the genus *Alouatta*. This phylogenetic proximity suggests a possible sharing of hemoplasmas among species with similar ecological niches. *Cyclopes*, *C. villosus*, and *Alouatta* spp. all exhibit strictly arboreal habits and inhabit humid tropical forests of South America, often with spatial overlap in different vegetation strata. Such ecological proximity may facilitate exposure to common arthropod vectors. The genetic similarity between hemoplasmas associated with these hosts, combined with their arboreal lifestyle, raises the hypothesis that these microorganisms may circulate among sympatric species with convergent ecological behaviors through transmission routes that remain poorly understood.

The actual clinical impact of hemoplasma infections in these hosts is still uncertain, as bacterial co-infections may or may not compromise the health of individuals [18]. These results reinforce the need for longitudinal monitoring to assess the effects of infection over time.

Behavioral aspects of arboreal mammals may contribute to parasitism by arthropods of vectorial importance. Sympatric nocturnal arboreal primates from Madagascar (*Avahi occidentalis* Lorenz, 1898, and *Lepilemur edwardsi* Forsyth Major, 1894) were evaluated for parasite exposure risk, exploring how the ecology of sleeping sites influences infestation by ectoparasites and vector-borne hemopathogens. It was observed that *L. edwardsi* (uses tree hollows, exhibiting strong roosting site fidelity), but not *A. occidentalis* (sleeps in open branches and frequently changes roosting site), harbored nest-adapted hard and soft ticks, as well as mites. The authors concluded that sleeping in tree cavities promotes ectoparasite infestation but may offer protection against hemopathogens transmitted by mosquitoes [81].

Silky anteaters exhibit arboreal and nocturnal habits and frequently change their sheltering sites. These behavioral characteristics may make them more susceptible to parasitism by hematophagous dipterans than by ectoparasites that need to remain attached to the host to feed on blood. Dipterans of the family Tabanidae are predominantly active during the day or at twilight [15,63], meaning they are most active when *Cyclopes* spp. are at rest. In this context, the inactivity of silky anteaters during peak vector activity may increase their exposure to bites, as the absence of defensive behaviors can facilitate vector access. Therefore, it is plausible that tabanid flies are involved in the transmission of *T. evansi* and hemoplasmas to *Cyclopes* spp.

Previous studies on Xenarthra have reported the presence of ectoparasites, including ticks of the genera *Amblyomma* spp. Koch, 1844, and *Rhipicephalus* spp. Koch, 1844, as well as various flea species [82,83,84,85]. However, consolidated records regarding ectoparasites in species of the genus *Cyclopes* remain scarce. Miranda et al. [86] reported the parasitism of a *C. didactylus* individual by an engorged nymph of the tick *Amblyomma humerale*, a species endemic to South America whose adult stages are commonly associated with tortoises such as *Chelonoidis denticulata* Linnaeus, 1766, and *C. carbonária* Spix, 1824. This finding suggests that species of the genus *Cyclopes* may serve as accidental or transient hosts for immature stages of *A. humerale*. However, the absence of ectoparasite detection in the present study prevented the correlation between the findings and potential vectors, highlighting the need for further investigations to better understand the relationship between *Cyclopes* spp. and ectoparasites, as well as their potential role in the maintenance and dispersal of these arthropods in natural environments. The limitation of the inspection method, restricted to macroscopic visual examination, may have contributed to the absence of ectoparasite records, considering that immature forms or small-sized specimens might not be detected without the use of auxiliary tools. More sensitive approaches, such as the use of entomological combs and fine forceps, are recommended in future studies to maximize ectoparasite recovery, especially in species with dense fur.

This study investigated vector-borne hemopathogens using samples obtained from a biological bank previously collected for the genetic analysis of the animals [1]. However, the limited volume of the samples restricted the amplification of additional gene regions in the positive samples, as well as the repetition of sequencing reactions that yielded suboptimal results. Furthermore, the preservation status of the samples, which had been stored frozen, precluded the application of other direct parasitological methods, such as blood smears, Woo’s technique, buffy coat analysis, and pathogen culture and isolation. The absence of morphological examination is recognized as a limitation, as it hinders the confirmation of active infections and may lead to underdiagnosis in cases of mixed infections, which are common in wildlife. Additionally, molecular detection alone may reflect transient DNA presence from recent vector inoculation, rather than true host infection or susceptibility.

In conclusion, silky anteaters were found to be parasitized by *T. evansi*, *T. cruzi*, and hemotropic *Mycoplasma*. This study highlights the need for future research to evaluate the potential impacts of these infections on the health of *Cyclopes* spp. The results presented here may serve as a foundation for more comprehensive investigations and support wildlife health managers and conservation authorities in the development of management strategies and public policies aimed at the conservation of this species.

## Figures and Tables

**Table 1:** Identification of the samples and geographical distribution of *Cyclopes* species included in this study.

Sample Identification	Species	Age	Location: Country/State/County
CD002	*C. didactylus*	Adult	Brazil/MA/São Luís
CD004	*C. didactylus*	Adult	Brazil/PE/Igarassu
CD005	*C. didactylus*	Adult	Brazil/PE/Timbaúba
CD006	*C. didactylus*	Adult	Brazil/PE/Jaboatão dos Guararapes
CD008	*C. didactylus*	Adult	Brazil/PA/Oriximiná
CD010	*C. didactylus*	Adult	Brazil/RN/Goianinha
CD011	*C. rufus*	Adult	Peru/Ucayali/Atalaya
CD012	*C. ida*	Adult	Peru/Ucayali/Purús
CD015	*C. didactylus*	Adult	Brazil/AM/Santa Isabel do Rio Negro
CD018	*C. ida*	Adult	Peru/Loreto/Maynas
CD022	*C. didactylus*	Adult	Brazil/MA/Rosário
CD026	*C. rufus*	Adult	Brazil/RO/Espigão do Oeste
CD030	*C. thomasi*	Adult	Brazil/AC/Porto Walter
CD032	*C. didactylus*	Adult	Brazil/AM/Manaus
CD034	*C. dorsalis*	Adult	Colombia/Santander/Girón
UFMG 6015	*C. rufus*	Adult	Brazil/RO/Porto Velho

**Table 2:** Description of primer sequences, target identification, and expected PCR amplicon sizes for hemopathogen detection.

Agents		Primer	Sequences (5’-3’)	Target	Fragment (bp)	Reference
Piroplasmida/ *Hepatozoon* spp. screening	1st reaction	RIB-19	CGGGATCCAACCTGGTTGATCCTGC	18S rRNA	1700	[22]
		RIB-20	CCGAATTCCTTGTTACGACTTCTC			
	2nd reaction	BAB-Rum F	ACCTCACCAGGTCCAGACAG	18S rRNA	430	[23]
		BAB-Rum R	GTACAAAGGGCAGGGACGTA			
Piroplasmida screening		HSP70F2	GGATCAACAAYGGMAAGAAC	*hsp70*	720	[24]
		HSP70R2	GBAGGTTGTTGTCCTTVGTCAT			
*Ehrlichia* spp. screening	1st reaction	N516SCH 1 F	ACGGACAATTGCTTATAGCCTT	16S rRNA	1195	[25]
		N516SCH 1 R	ACAACTTTTATGGATTAGCTAAAT			
	2nd reaction	N516SCH 2 F	GGGCACGTAGGTGGACTAG	16S rRNA	443	[25]
		N516SCH 2 R	CCTGTTAGGAGGGATACGAC			
*Anaplasma* spp. screening	1st reaction	GE3a	CACATGCAAGTCGAACGGATTATTC	16S rRNA	932	[26]
		GE10R	TTCCGTTAAGAAGGATCTAATCTCC			
	2nd reaction	GE9F	AACGGATTATTCTTTATAGCTTGCT	16SrRNA	546	[26]
		GE2	GGCAGTATTAAAAGCAGCTCCAGG			
*Anaplasma phagocytophilum* characterization	1st reaction	MSP4AP5	ATGAATTACAGAGAATTGCTTGTAGG	*msp4*	849	[27]
		MSP4AP3	TTAATTGAAAGCAAATCTTGCTCCTATG			
	2nd reaction	msp4F	CTATTGGYGGNGCYAGAGT	*msp4*	450	[28]
		msp4R	GTTCATCGAAAATTCCGTGGTA			
Hemotropic *Mycoplasma* screening/characterization		HBT F	ATACGGCCCATATCCCTACG	16S rRNA	600	[29]
		HBT R	TCGCTCCACCACTTGTTCA			
Hemotropic *Mycoplasma* screening		HemF	ACGAAAGTCTGATGGAGCAATA	16S rRNA	170–193	[30]
		HemR	ACGCCCAATAAATCCGRATAAT			
Hemotropic *Mycoplasma* characterization		23S_HAEMO_F	TGA GGG AAA GAG CCC AGA C	23S rRNA	800	[31]
		23S_HAEMO_R	GGA CAG AAT TTA CCT GAC AAG G			
*Anaplasma marginale* screening/characterization	1st reaction	MSP45	GGGAGCTCCTATGAATTACAGAGAATTGTTTAC	*msp4*	872	[32]
		MSP43	CCGGATCCTTAGCTGAACAGGAATCTTGC			
	2nd reaction	AnapF	CGCCAGCAAACTTTTCCAAA	*msp4*	294	[33]
		AnapR	ATATGGGGACACAGGCAAAT			
Kinetoplastida screening	1st reaction	SSU450F	TGGGATAACAAAGGAGCA	18S rRNA	928–1984	[34]
		SSU450R	CTGAGACTGTAACCTCAAAGC			
	2nd reaction	TRY927F	CAGAAACGAAACACGGGAG	18S rRNA	927	[34]
		TRY927R	CCTACTGGGCAGCTTGGA			
Kinetoplastida screening/characterization		TCZ1	CGAGCTCTTGCCCACACGGGTGCT	*kDNA*	180	[35]
		TCZ2	CCTCCAAGCAGC GGATAGTTCAGG			
*Trypanosoma evansi* screening/characterization	1st reaction	Te1F	GCACAGTATGCAACCAAAAA	ITS	280	[36]
		Te1R	GTGGTCAACAGGGAGAAAAT			
	2nd reaction	Te2F	CATGTATGTGTTTCTATATG	ITS	219	[36]
		Te1R	GTGGTCAACAGGGAGAAAAT			
*Trypanosoma vivax* screening/characterization		DTO154	ACAGAATTCCAGGGCCAATGCGGCTCGTGCTGG	Catepsin L	500	[37]
		DTO155	TTAAAGCTTCCACGAGTTCTTGATGATCCAGTA			
*Trypanosoma cruzi/* *Trypanosoma rangeli* screening/characterization		S35	AAATAATGTACGGGKGAGATGCATGA	*kDNA*	330	[38]
		S36	GGTTCGATTGGGGTTGGTGTAATATA			
*Leishmania* spp. screening/characterization		LITSR	CTGGATCATTTT CCGATG	ITS1	300–350	[39]
		L5.8S	TGATACCACTTA TCGCACTT			
*gapdh*		GAPDH F	CCTTCATTGACCTCAACTACAT	*gapdh*	400	[21]
		GAPDH R	CCAAAGTTGTCATGGATGACC			

**Table 3:** Hemopathogen infections detected in 16 silky anteaters (*Cyclopes* spp.) from Brazil, Peru, and Colombia.

Sample Identification	Country	Agent							
		*T. evansi*	*T. cruzi*	*T. vivax*	*Leishmania* spp.	*Mycoplasma* spp.	Piroplasmida/*Hepatozoon* spp.	*Ehrlichia* spp.	*Anaplasma* spp.
CD002 *C. didactylus*	Brazil	Positive	-	-	-	-	-	-	-
CD004 *C. didactylus*	Brazil	Positive	-	-	-	-	-	-	-
CD005 *C. didactylus*	Brazil	-	-	-	-	Positive	-	-	-
CD006 *C. didactylus*	Brazil	-	-	-	-	Positive	-	-	-
CD008 *C. didactylus*	Brazil	Positive	-	-	-	Positive	-	-	-
CD010 *C. didactylus*	Brazil	-	-	-	-	Positive	-	-	-
CD011 *C. rufus*	Peru	-	Positive	-	-	Positive	-	-	-
CD012 *C. ida*	Peru	Positive	-	-	-	Positive	-	-	-
CD015 *C. didactylus*	Brazil	-	-	-	-	-	-	-	-
CD018 *C. ida*	Peru	Positive	-	-	-	Positive	-	-	-
CD022 *C. didactylus*	Brazil	-	-	-	-	Positive	-	-	-
CD026 *C. rufus*	Brazil	Positive	-	-	-	-	-	-	-
CD030 *C. thomasi*	Brazil	Positive	-	-	-	Positive	-	-	-
CD032 *C. didactylus*	Brazil	Positive	-	-	-	-	-	-	-
CD034 *C. dorsalis*	Colombia	-	-	-	-	-	-	-	-
UFMG 6015 *C. rufus*	Brazil	-	-	-	-	Positive	-	-	-

Negative (-).

## Data Availability

Data are contained within the article.
